# Quality improvement collaborative approach to COVID-19 pandemic preparedness in long-term care homes: a mixed-methods implementation study

**DOI:** 10.1136/bmjoq-2023-002589

**Published:** 2024-04-08

**Authors:** Janice Sorensen, Laura Kadowaki, Lucy Kervin, Clayon Hamilton, Annette Berndt, Simran Dhadda, Abeera Irfan, Emma Leong, Akber Mithani

**Affiliations:** 1 Long‐Term Care and Assisted Living, Fraser Health Authority, Surrey, British Columbia, Canada; 2 Simon Fraser University Gerontology Research Centre, Vancouver, British Columbia, Canada; 3 Simon Fraser University, Burnaby, British Columbia, Canada; 4 Long‐Term Care and Assisted Living Research Partners Group, Fraser Health Authority, Surrey, British Columbia, Canada; 5 Department of Psychiatry, The University of British Columbia, Vancouver, British Columbia, Canada

**Keywords:** Implementation science, COVID-19, Long-Term Care, Quality improvement, Nursing homes

## Abstract

**Background:**

The devastating impact of the COVID-19 pandemic on long-term care (LTC) homes underscores the importance of effective pandemic preparedness and response. This mixed-methods, implementation science study investigated how a virtual-based quality improvement (QI) collaborative approach can improve uptake of pandemic-related promising practices and shared learning across six LTC homes in British Columbia, Canada in 2021 during the COVID-19 pandemic health emergency.

**Methods:**

QI teams consisting of residents, family/informal caregivers, care providers and leadership in LTC homes are supported by QI facilitation and shared learning through virtual communication platforms. QI projects address gaps in outbreak preparation, prevention and response; planning for care; staffing; and family presence. Thematically analysed semi-structured qualitative interviews and a validated questionnaire on organisational readiness investigated participants’ perceptions of challenges, success factors and benefits of participating in the virtual QI collaborative approach.

**Results:**

Nine themes were identified through interview analysis, including two related to challenges (ie, making time for QI and hands tied by external forces), four regarding factors for successes (ie, team buy-in, working together as a team, bringing together diverse perspectives and facilitators keep us on track) and three on the benefits of the QI collaborative approach (ie, seeing improvements, staff empowerment and appetite for change). Continuous QI facilitation and coaching for QI teams was feasible and sustainable virtually via video conferencing (Zoom). The QI team members showed limited engagement on the virtual communication platform (Slack), which was predominantly used by the implementation science team and QI facilitators to coordinate the study and QI projects, respectively.

**Conclusions:**

The virtual-based QI collaborative approach to pandemic preparedness supported LTC homes to rapidly and successfully form multidisciplinary QI teams, learn about QI methods and conduct timely QI projects to implement promising practice for improved COVID-19 pandemic response.

**WHAT IS ALREADY KNOWN ON THIS TOPIC**

- Quality improvement (QI) collaboratives are widely used to improve healthcare but there is limited evidence on effective QI approaches in long-term care (LTC) and pandemic circumstances.

**WHAT THIS STUDY ADDS**

- A virtual QI collaborative approach can indeed be successful in LTC settings and a pandemic health emergency context. A flexible approach is needed due to the limited time of staff and varied, dynamic needs of LTC homes in pandemic times, which necessitates LTC-adapted QI tools and supports.

**HOW THIS STUDY MIGHT AFFECT RESEARCH, PRACTICE OR POLICY**

- QI infrastructure can aid in conducting timely QI projects by LTC homes, and in circumstances where in-person communication is not feasible. Exploring alternative methods to foster communication within QI collaboratives is essential and should be considered in future research.

## Introduction

The COVID-19 pandemic disproportionally impacted residents in long-term care (LTC) homes with increased rates of infections, poorer outcomes and death.^1 2^ Although this could be attributed to resident-related factors, such as older age and comorbidities, several modifiable factors for improvement have been identified.^3^ These factors include: COVID-19 outbreak preparation, prevention, response and surge capacity; care plans; workforce; and family presence in LTC homes.^3^ The risk of outbreaks in LTC has made quality improvement (QI) for pandemic preparedness and response a top priority. This has been supported by Healthcare Excellence Canada (HEC) initiatives aimed to implement promising practices and policies in LTC homes across Canada to improve outcomes.^4^

Different approaches to QI should be considered for optimal support in pandemic preparedness in LTC. Such approaches should address challenges faced in this unique context. For example, physical distancing guidelines restricted presence of key QI team members in LTC homes, including family/informal caregivers impacted by visitation restrictions and physicians and other healthcare providers that shifted to telehealth. Relevant to this challenge, virtual adaptation of traditional QI training, learning resources and means of communication holds promise. Various QI approaches reported at the start of the COVID-19 pandemic included increased frequency of QI meetings/huddles; integrated structure and function of QI committees and creation of task forces in addition to virtual adaptations.^5 6^ Another approach is QI collaboratives, which involve collective QI supports and shared learning on a topic for rapid improvement across multiple sites.^7 8^ QI collaboratives have been successfully applied in groupings of LTC homes internationally, in other pandemic/health emergency contexts, and involving virtual adaptations.^9–12^

This PAandemic Preparedness in LOng-term Care Homes (PAPLOC) study is part of the ‘Implementation Science Teams (IST): Strengthening Pandemic Preparedness in LTC’ programme of HEC in 2021.^13^ The PAPLOC study explores how a virtual-based QI collaborative approach in LTC homes can support implementation of promising practices and shared learning for pandemic preparedness.

## Methods

### Setting

Fraser Health Authority (FHA) is a publicly funded healthcare region in British Columbia (BC) delivering hospital and community-based health services to the most populated health region in BC with over 1.9 million people. It includes 7760 publically funded LTC beds across 81 LTC homes. All LTC site managers and leaders in the FHA region were invited to take basic QI training based on the curriculum of the Institute for Healthcare Improvement (IHI). Those who participated in this training were extended an invite for their LTC home to take part in the study, in which six LTC homes participated.

### Conceptual framework

The Consolidated Framework for Implementation Research (CFIR)^14^ comprised the study’s conceptual foundation informing design, data collection and analysis. CFIR^14^ can guide systematic assessment of multilevel implementation contexts and identification of factors potentially impacting implementation and effectiveness of complex interventions in care delivery settings.^15 16^ Its constructs span five domains (ie, Intervention Characteristics, Outer Setting, Inner Setting, Characteristics of Individuals and Process) most likely to influence intervention implementation.^14^ Application of the CFIR^14^ enabled comprehensive investigation of multiple factors relevant to intervention implementation in the LTC setting and pandemic context.

### Intervention description

The PAPLOC study investigated a virtual QI collaborative approach for implementing promising practices and policies for pandemic preparedness in LTC. A detailed description can be found in table 1.

**Table 1:** Description of the intervention*

Brief name	PAndemic Preparedness in LOng-term Care Homes (PAPLOC)
Why	Aimed to rapidly support LTC homes to identify, implement and share promising practices and policies for COVID-19 pandemic preparedness, including COVID-19 outbreak preparation, prevention, response and surge capacity; COVID-19/non-COVID-19 care plans; strengthening the workforce and family presence in LTC homes^27^
What	*Formation of QI teams*: each LTC home formed a multidisciplinary QI team (~5–8 members) comprised of nurses, physicians, allied health professionals, managers/leaders and (encouraged) residents and family/informal caregivers. *QI facilitation*: an initial meeting was held with each QI team where a QI facilitator introduced the PAPLOC approach. At the next meeting, the QI facilitator reviewed a self-assessment^27^ with the QI team to identify pandemic-related change ideas. Subsequent meetings involved facilitated development of aim statements for the teams’ selected QI projects, performance of project Plan-Do-Study-Act (PDSA) cycles and documentation of findings. The QI facilitators used LTC-adapted tools to support QI in three LTC homes each. QI teams were coached to start with simple QI projects. QI training was provided based on the *Model for Improvement* by associates in process improvement. *Collaborative activities*: shared learning involved collaborative activities, for example, newsletters and facilitated lesson sharing supported by the QI facilitators. *Virtual collaborative communication platform*: Slack, a cloud-based communication platform with direct and group synchronous and asynchronous messaging, audio and video calling, was used for communication and sharing information with the QI teams. Team members were given guidelines and resources on use of the platform and access to one-on-one support.
Who provided	Two QI facilitators (a physician lead in clinical quality and research in LTC and assisted living and a clinical nurse specialist in clinical quality and patient safety) and one QI facilitation assistant (SD) who were all trained in the QI curricula of the Institute for Healthcare Improvement (IHI)
How	Virtual meetings between the QI facilitators and QI teams (ie, video conferencing via Zoom), except for one in-person meeting at one LTC home
Where	Six LTC homes in mid-size or major population centres in the FHA region
When and how much	Eight months (March 2021 to November 2021) with weekly or bi-weekly meetings between the QI facilitators and QI teams
Tailoring	The intervention was conceptualised as flexible to accommodate for the LTC homes that varied in QI experience, capacity and COVID-19 pandemic status. It was adapted throughout the study to meet the needs of specific LTC homes regarding: (a) the number of QI team members and their roles; (b) the method of delivery for team meetings (ie, a face-to-face meeting took place with one team); (c) the number and complexity of QI projects underway and (d) the pace at which QI teams progressed through the QI facilitation and PDSA cycles dependent on their experience, capacity and the specific QI projects.
Modifications	The QI teams were originally planned to consist of the same members throughout the full intervention period. Based on feedback from the QI facilitators in consideration of the dynamic nature of QI, a core team was formed at each LTC home and additional team members were brought on as appropriate to assist with specific projects. The Slack virtual platform was originally planned to be used as the primary means of communication within and across QI teams. During the course of the intervention, barriers to using the platform were experienced (eg, lack of time, accessibility issues for resident team members with physical disabilities, care staff with limited access to computers on site, etc). Consequently, other means of communication (eg, Zoom video conferencing, email) were used more frequently. Across homes, collaboratives activities for shared learning was achieved through sharing information about the completed, ongoing and planned QI projects in newsletters and through dedicated discussion periods with QI facilitators.
How well	Although intervention adherence or fidelity was not directly assessed, the QI facilitators and IST communicated continuously in weekly meetings to support consistency of the approach across LTC homes and note any modifications. Barriers to implementing the intervention related to internal processes in the LTC homes (ie, changes in leadership, staffing shortages, workload) and external factors, which exacerbated the difficult pandemic circumstances (eg, dangerous heat dome in the region, accommodating evacuee LTC residents due to wildfires in adjacent regions). These barriers did not negatively impact the QI, but did impact the timelines.

*The description of the intervention is based on the Template for Intervention Description and Replication (TIDier) checklist.

LTC, long-term care; QI, quality improvement.

### Participants and data collection

All QI team members were eligible and invited to participate in the study via email. A series of semi-structured interviews (baseline, midpoint and final) were conducted with participants from April to October 2021, with an approximate 6–10 weeks gap between each interview. Out of 25 total study participants, 24 completed the baseline interview, 12 the midpoint interview and 22 the final interview. Reasons for incomplete interviews included challenging circumstances at the LTC homes (eg, staffing shortages, severe weather events over the summer) and at one LTC home, administrative challenges arranging back-pay for staff. Six people declined to participate in the interviews for various reasons (eg, lack of time), but did participate in the QI collaborative.

Following verbal consent from participants, authors L Kadowaki and L Kervin, research coordinators trained in qualitative methods, conducted the semi-structured interviews from March to October 2021. Interviews conducted over Zoom or the telephone, as per public health distancing guidelines, ranged from 20 to 60 min in length and followed a guide consisting of open-ended and close-ended questions (see online supplemental file 1). The interviews were audio-recorded and transcribed verbatim.

10.1136/bmjoq-2023-002589.supp2 Supplementary data

For participants employed in the LTC homes, the Organisational Readiness for Knowledge Translation (OR4KT) questionnaire^17^ was administered during the baseline and final interviews. This validated questionnaire consists of 59 statements within six domains: organisational climate for change, organisational contextual factors, change content, leadership, organisational support and motivation. Participants responded to the statements using a 5-point Likert scale where 1 is ‘strongly disagree’ and 5 is ‘strongly agree’.

### Data analysis

Thematic analysis is a common qualitative data analysis approach that finds patterns within data and highlights the most salient aspects of the dataset.^18 19^ The codebook approach, a type of thematic analysis, was used for interpretation of the interview data due to its structured nature and acknowledgement that there are many realities and that meaning relies on context, the underlying philosophy of qualitative reflexivity.^18^ This data analysis approach allows a team of researchers to easily work together on the analysis and for the replication.^18 20^ A hybrid inductive-deductive approach was applied for the codebook development, involving the a priori creation of a list of expected codes based on the interview questions, insights from the interviews and the addition of emergent codes that arose from the coding process. During the theming process, the themes were mapped out to the CFIR^14^ constructs to structure their presentation and interpretations. This approach allows researchers to leverage pre-existing knowledge, while also recognising the possibility of emerging concepts from the data.^19 21^ Hybrid inductive-deductive approaches to codebook-based thematic analysis have been used in past research.^20 22^

The IST collectively reviewed a randomly selected transcript to pilot the developed codebook. During the coding process, additional codes that emerged from the data were added to the codebook. The analysis process consisted of five rounds: (a) initial coding of the transcripts based on the codebook, (b) review of the coding by a second coder, (c) identification of themes from the coded data, (d) review of the themes with the IST members including a family/caregiver partner (AB) and (e) member checking with study participants. Member checking involved sending a summary of themes to participants for feedback and offering them the option to provide feedback; no feedback was received. The NVivo V.12 program was used for qualitative data coding as it allows for organisation and in-depth analysis.

The OR4KT data was analysed by descriptive statistics for the individual items and cumulatively for the five domains. Only data from participants who completed both a baseline and a final interview were retained for the analysis (n=22). Findings from the OR4KT data were used to support the insights gathered from interviews.

### Patient and public involvement

The IST included an LTC family/caregiver partner (AB) who contributed to development of the PAPLOC approach, study conduct, including participation in weekly IST meetings, qualitative analysis, and dissemination, for example, review of newsletters and this manuscript. Family and resident partners recruited into the QI teams assisted with identification of site-level priorities and conducting QI projects. Project achievements were shared with these partners via newsletters and published manuscripts will also be circulated.

## Results

### Participant demographics

25 QI team members across the six LTC homes participated in the study. Most participants were female (84%), aged between 50 and 59 years (36%), a manager or site leader (32%), identified as white (56%), and university-educated (48%) (table 2).

**Table 2:** Participant demographics (n=25) and long-term care (LTC) home characteristics (N=6)

Characteristics	Number (n)	Frequency (%)
Gender		
Female	21	84
Male	3	12
Not disclosed	1	4
Age		
30–39	3	12
40–49	7	28
50–59	9	36
60 or older	4	16
Not disclosed	2	8
Racial/cultural identification		
White	14	56
Asian	6	25
Mixed	1	4
Not disclosed	4	16
Role in LTC		
Resident	2	8
Family of resident/informal caregiver	1	4
Nurse*	6	16
Physician	3	12
Allied health professional	5	20
Site manager/leader	8	32
Level of education (initiated or completed)		
High school	1	4
College diploma	4	16
University degree	12	48
Masters/postgraduate degree	7	28
Not disclosed	1	4
LTC home operator type		
Public	1	17
Private for-profit	2	33
Private not-for-profit	3	50
LTC home size (number of beds)		
Less than 50	1	17
50–100	2	33
100–150	3	50

*‘Nurse’ includes registered nurses (RN) and licensed practical nurses (LPN).

### Interview findings: themes on overall QI collaborative approach

The nine main themes identified through analysis of the baseline, midpoint and final interviews are described below. Two of the themes pertain to challenges that influenced implementation and participation in the QI collaborative, which relate to both the CFIR Inner Setting and Outer Setting domains.^14^ Four themes are success factors for the QI collaborative involving the CFIR domains of Inner Setting, Process and Characteristics of Individuals.^14^ Finally, three themes on the benefits of the QI collaborative relate to the CFIR Inner Setting and Process domains.^14^ Table 3 contains representative quotes for each theme and related CFIR domains and constructs.^14^

**Table 3:** Themes on the QI collaborative approach and representative quotes

Theme	Representative quotes	Related CFIR constructs
Making time for QI	Time. I think time is the barrier. (B1) It was a good experience, something I hadn’t been involved with before. I wish I had more time. You know, to be more and more involved, you know, with meetings or just moving forward. (F15)	**Readiness for implementation (Inner Setting domain):** the QI collaborative was affected by the time LTC homes had for QI and ongoing operations
Hands tied by external forces	I think if it had been a year ago, or even like two years ago, and we weren’t talking about COVID, but we were just talking about QI projects, I know we would all have more energy, right, and more focus. (F4) I mean, I think everybody’s doing their best. It’s so, it changes so fast. Like every single day, it seems something changes. (F5)	**External policies and incentives (Outer Setting domain):** external policies, challenges and factors related to the COVID-19 pandemic served to hinder the implementation of the QI collaborative
Team buy-in	The staff are reluctant for change so anytime we come up with something, it’s all about the presentation and how you give it to them, whether or not you’re going to have much buy-in from the staff. I think if it’s presented without their input so if it’s, so if it’s deemed as coming from the top down, you don’t get as much buy-in. (B19) Everybody is buying into it and doing this work, I think that’s a thing that makes me very satisfied. (F17)	**Implementation climate (Inner Setting domain):** team buy-in was enhanced by perceived importance of the QI collaborative resulting in improvements in the LTC homes **Knowledge and beliefs about the intervention/self-efficacy (Characteristics of Individuals domain):** individual buy-in was enhanced by the positive attitudes of team members towards QI and belief in their ability to affect change
Working together as a team	Yeah, I think that the team working on it together. So, just the—some of the staff, the way they developed the communication tool and adapted it from previous, as well as the nurses engaging with education. So, I think that the teamwork together has been what’s allowed things to be successful. (F1) So because like during our meeting, so everyone has input and then—so it’s a collaborative—it’s a collaboration with everyone that’s involved with the QI project I think that’s what made it a success. (M8)	**Implementation climate (Inner setting domain):** QI teams were highly receptive to the intervention and there was a positive learning climate where team members felt everyone was valued and had opportunities to contribute
Bringing together diverse perspectives	I appreciate that everybody who’s on the team brings their own personal expertise and that they’re so willing to share it with everyone else and empower the rest of us. I think that’s, that’s very beneficial and appreciated by everyone here at [name of care home]. (M10) I understand their(my colleagues’)struggles, they understand my struggles. We feel, I feel closer to them. I feel that I can respect their work and they can respect my work better. (M17)	**Engaging (Process domain):** QI teams were able to attract and involve a wide variety of members, including managers, health providers, residents and family/caregivers
Facilitators keep us on track	It was(a)really great opportunity to kind of methodically look at issues and you know, break them down into manageable cases. And you know, having that supportive group to kind of problem solve through little road blocks and that sort of thing. (F7) I think they [QI facilitators] keep us on track. I think they keep us focused so that we don’t wander and try and do too much at one time. So, they centre us. (F21)	**Planning, engaging and executing (Process domains):** QI facilitators acted as external change agents and played key roles in project planning with the QI teams, ensuring all members were engaged, and assisting in conducting the PDSA cycles
Seeing improvements	Because there—I’ve seen the difference before we did the QI projects that we have, there were a lot of concerns and there were a lot of questions and then once the QI was implemented it was a breeze and family members are actually—we’ve had family members commenting that it helped a lot once we implemented that project. (M8) A lot of the things that we’ve talked about, and that we’ve implemented, and that we’ve gone through have been really important for not only for staff, but for residents. So, it’s been just a very positive group, and I’m very happy to be a part of it, you know, seeing the changes that we have made or are trying to make, and the changes and happiness in the residents. (F14)	**Reflecting and evaluating (Process domain):** the opportunity for participants to see changes in real time through anecdotal evidence and collected data positively influenced their perceptions and buy-in
Staff empowerment	Because we are not just trying to tread water for the day-to-day, we are actually having a shared vision of how to improve things and, and seeing those improvements unfold. And even more importantly, being the drivers of that change. We get to decide what is important. (F17) It’s very enjoyable for me because, like communicating and telling them what is going on has already changed the quality of care with the residents. (M20)	**Reflecting and evaluating (Process domain):** over time, staff were able to observe changes in their own confidence and ability to affect change in the LTC home
Appetite for change	Well, I think in long-term care, there is a huge focus on how are we going to deliver services to our residents and their families in a different way? I really don’t think that what we’ve been doing in the past can work in the future. I think those days, I hope have changed, I hope that we’re able to grab the turmoil that has happened because of COVID and make some desperately needed changes. (F5) I don’t think that we can really look at it as complete or think about it as the completion of a study. I think it’s something that, you know, we’ve started, and we just have to keep moving forward with it. (F10)	**Reflecting and evaluating (Process domain):** participants reflected on the need for broader systemic changes in LTC homes **Implementation climate (Inner Setting domain):** there was tension for change and the staff perceived the need to make changes to improve care for residents and processes for staff

The CFIR domains (constructs) are: Intervention Characteristics (intervention source, evidence strength and quality, relative advantage, adaptability, trialability, complexity, design quality and packaging, and cost); Outer Setting (patients’ needs and resources, cosmopolitanism, peer pressure, and external policies and incentives); Inner Setting (structural characteristics, networks and communications, culture, implementation climate, readiness for implementation); Characteristics of Individuals (knowledge and beliefs about the intervention, self-efficacy, individual stage of change, individual identification with organisation, other personal attributes); and Process (planning, engaging, executing, reflecting and evaluating).

CFIR, Consolidated Framework for Implementation Research; LTC, long-term care; PDSA, Plan-Do-Study-Act; QI, quality improvement.

### QI challenges

*Making time for QI* was the most prominent challenge to participating in the QI collaborative. At baseline, QI was perceived by participants as an activity that they had to make time for among their other duties. This theme also emerged in the midpoint/final interviews, with participants stating that it was challenging to find time to participate in the QI collaborative or that they were not able to participate as much as they would have liked due to time constraints. Heavy workloads and staffing shortages contributed to this challenge. Time was also the most identified barrier for continuing to work on QI following the study.

The second theme related to participants’ experience of their *hands tied by external forces*. At baseline, midpoint and final interviews participants identified external forces that inhibited their ability to participate in QI. In the midpoint/final interviews, participants identified external forces related to the COVID-19 situation (eg, changing policies and restrictions, increased workload) and other emergencies (eg, climate/natural disasters) that hindered their QI participation. Some participants commented on the timing of the intervention, stating that they wished that it could have been implemented at another time when they were not struggling with compounding crises. Participants also voiced how the dynamic nature of the pandemic made it difficult to plan and implement changes. For example, one QI team had been developing an online visitation booking system consistent with provincial regulations that were dropped before the project could be completed. A few participants noted that the lack of external supports to face these emergencies had exacerbated the challenges.

### QI success factors

Three themes regarding the team-based nature of the intervention emerged in the interviews. At baseline, *team buy-in* was initially identified as a critical success factor for QI. At midpoint/final interviews, participants identified two additional success factors: *working together as a team* and *bringing together diverse perspectives. Team buy-in*, team members’ understanding, acceptance, support and agreement with the change, emerged as a key success factor at baseline, midpoint and final interviews. Most of the comments on team buy-in at midpoint/final interviews referred to buy-in of QI team members rather than of others who may have been affected by the QI changes in the LTC homes. Participants were proud of the commitment and enthusiasm of their QI teams and noted this increased their own enthusiasm for QI. For example, several members of one QI team spoke about being inspired by a fellow team member who had taken the initiative on her own time to make a poster on COVID-19 vaccines and speak with other staff members about the importance of being vaccinated.

*Working together as a team* was identified as a success factor in the midpoint/final interviews. Advantages of working collaboratively on QI included new ideas and the ability to share the work. The majority of participants believed that each team member contributed to the projects and were given opportunities to provide input. Nevertheless, a few participants pointed out the need to involve additional team members, especially care aides, or to distribute the workload more fairly. During the midpoint/final interviews *bringing together diverse perspectives* was identified as one of the most valuable aspects of the QI collaborative. Having a diverse QI team representing various groups within the home (eg, physicians, nurses, residents, family members, allied health, etc) was perceived as important. Participants described how they benefited from hearing the perspectives of other team members, which led to new understandings of issues and also of each other, improved communication with other groups, and resulted in new ideas.

A fourth theme was identified relating to the role of the QI facilitators, with participants describing how the *facilitators keep us on track*. At baseline, most participants conveyed that they had engaged in QI informally in the past and could see the value of doing QI in a more formal and structured way. They reported needing tools and supports to facilitate changes. In the midpoint/final interviews, participants reported on the benefits of having the QI facilitators available to provide the teams coaching to ‘keep on track’. The QI facilitators were described as bringing structure, consistency, and accountability to QI efforts. Participants identified the most needed resource for future QI work as continued facilitation support (with desired levels of support ranging from an as-needed basis to regular facilitation and meetings).

### Benefits of participating in QI collaborative

Three themes emerged regarding the perceived benefits of participating in the QI collaborative: *seeing improvements*, *staff empowerment* and *appetite for change*.

Being able to *see improvements* resulting from the QI projects had a powerful impact on participants. At baseline, participants had identified QI as a valuable activity that could improve processes and quality of care for residents. In the midpoint/final interviews, perceptions of the QI initiatives and collaborative were positively impacted by the ability of participants to see progress on QI projects and improvements in their LTC home. Even before QI projects were done, participants felt a sense of satisfaction from being able to see small real-time improvements as a result of their projects.

In the midpoint/final interviews, *staff empowerment* emerged as an additional benefit of the QI collaborative. Participants expressed a sense of satisfaction and empowerment from participating in the QI collaborative. Some participants specifically commented on the importance of having their voice heard and feeling like they were able to make a difference. Several participants stated they felt more confident after participating in the QI collaborative. In particular, participants at LTC homes that implemented ongoing/lengthier QI projects expressed a sense of empowerment.

Finally, the theme *appetite for change* was prominent at baseline and persisted at the midpoint/final interviews. At baseline, participants expressed an appetite for change, believing there was a need for QI in LTC and expressing enthusiasm for the QI approach. At the midpoint/final interviews, having seen the positive results of their QI projects, as well as lingering issues, participants expressed a strong desire to continue to work on QI after the study ended. Some participants also wanted to see more big-picture changes, noting that while some improvements had been made in pandemic preparedness, they really only scratched the surface and the need for further QI was clear.

### Interview findings: virtual components of the QI collaborative approach

A key component of the PAPLOC approach was using a virtual platform to deliver the QI collaborative. As such, participants were asked about their perspectives and engagement with the virtual components of PAPLOC at baseline, midpoint and final interviews. Comments regarding the virtual component during interviews transpired in response to specific questions but proved insufficient to establish an overarching theme. However, due to the prevalence of certain responses, such as a preference for in-person QI over virtual methods, these responses were quantified through supplementary analysis of the interview data.

At baseline, participants discussed how COVID-19 was a game-changer for the use of technology, and how since the pandemic, there has been an increased openness to using technology and how technology has become embedded in their daily practices. The virtual communication platforms that participants were most familiar with and used were Zoom (79%) and Microsoft Teams (63%). Participants highlighted the convenience of technology for holding meetings and communicating, but also noted potential challenges related to technical difficulties and lack of digital literacy. Half the participants still preferred to communicate face-to-face, while the other half preferred hybrid (virtual and face-to-face) (46%) or virtual (4%) communication. Overall, participants expressed an openness to using virtual collaborative tools.

In the midpoint/final interviews, participants reported ambivalent feelings about the virtual collaborative and communication tools (Slack and Zoom) that were used. Experiences with Zoom were generally positive, and participants found it to be an effective platform for holding team meetings with the QI facilitators:

I do prefer in-person so we can work on some of the graphs and, and things, but being a pandemic and all, I was pleasantly surprised by how much we accomplished through online meetings. And I think it allowed us to actually have more meetings with people off-site, because we wouldn’t have probably had the support of [QI facilitator] if she had to drive in every time, you know, for an hour meeting. (F17)

However, several participants expressed dissatisfaction with Zoom and preferred that the first and occasional meeting or all meetings be held in-person.

The majority of participants joined Slack early in the study and generally expressed positive views of its use. However, engagement on Slack remained minimal throughout the intervention, apart from the QI facilitators who used Slack for sharing QI documents. In the interviews, a variety of factors emerged that impacted participants’ utilisation of Slack (table 4). The most prominent of these factors was the availability of other more convenient means of communication, such as face-to-face communication because most team members were generally on-site together.

**Table 4:** Factors contributing to low utilisation of the virtual collaborative communication platform (Slack)

Factors contributing to low utilisation of Slack	Representative quotes
**Other means of communication:** many participants perceived that there were other more convenient ways to communication with their QI team including face-to-face for on-site team members, Zoom, email and/or text	I think because the process that we have, we communicate with our team here in the site. We are in-person, we are going into the meetings. (F23)
**Too many platforms:** some participants expressed feeling fatigued or overwhelmed by the many online platforms (eg, Zoom, Microsoft Teams, Slack) being introduced and used for communication during the pandemic	So why do we need a whole new platform to do what we are already doing, I don’t know. (M5)
**Difficulties using the platform:** a small number of participants reported technical difficulties when using Slack as a barrier	No, actually, I tried to follow the instructions you sent me about how to participate in the Slack. And then I tried to open it, but it doesn’t let me get through. (F20)
**Lack of time:** some participants reported a lack of time to learn/use the platform	Yeah, it’s just—well, you know, I think, again, it’s a great tool but when things are so busy, it’s hard to then be mindful of checking, like, another platform for updates. (F1)
**Lack of accessibility of Slack:** accessibility issues were barriers to the use of Slack for participants who (a) did not have access to appropriate devices or (b) had a disability that impacted their ability to use the platform	Well, our challenge was trying to get (resident partner) involved, and we couldn’t get him set up by himself. (F4)

### Organisational Readiness for Knowledge Translation

Based on the overall distribution of responses (see online supplemental table 1), the OR4KT dimensions appraised most favourably at baseline data (n=22) were *organisational climate for change* (76%), *leadership* (73%), *organisational contextual factors* (73%) and *organisational support* (70%). Only these 4/6 dimensions (ie, excluding *change content* and *motivation*) were scored positively by more than 2/3 of respondents. At intervention completion (final; n=22), the most favourably appraised dimensions were *organisational climate for change* (87%), *organisational support* (82%), *organisational contextual factors* (81%) and *change content* (80%). All six dimensions received a favourable final scored by more than 2/3 of respondents.

10.1136/bmjoq-2023-002589.supp1 Supplementary data

Specific questionnaire statements were noted to reflect the themes that arose in the qualitative interviews. Aligning with the theme *working together as a team*, 100% of respondents agreed with the statement that staff work together as a team at final interview, increasing from 73% at baseline. The theme of *staff empowerment* was evident in that 96% of respondents at midpoint agreed that staff members feel free to ask questions and express concerns and that managers are open to staff ideas for improving change, increasing from 77% at baseline. Aligning with the themes *team buy-in* and *appetite for change*, respondents agreeing at final interview that team members provide practical support for new ideas and their application (96%) and that pressure to make change comes from staff members (55%) increased from 73% and 27% at baseline, respectively. The theme *hands tied by external forces* was evident in that 55% of respondents agreed at final interview that their organisation had necessary support in terms of staffing numbers to facilitate change, decreasing from 73% at baseline. Reinforcing the themes *facilitators keep us on track* and *seeing improvements*, respondents agreeing at intervention completion that assistance in developing new ideas is readily available (77%) and that the change process is monitored continuously (77%), increasing from 64% and 50% at baseline, respectively.

## Discussion

This is the only implementation science study to our knowledge investigating a virtual QI collaborative approach for pandemic preparedness in LTC during a pandemic. In contrast to most studies conducted in LTC during the COVID-19 pandemic showing substantial gaps in practice, vulnerability and/or poor outcome,^1 2^ this study found that implementation of the PAPLOC virtual QI collaborative approach was possible in LTC and related to teamwork, inclusion, empowerment and appetite for change. Further, QI projects led to advancements across the six LTC homes within a short period during the pandemic. This work also provides insights on challenges, success factors and benefits of the PAPLOC approach, which could inform future best practices for virtual QI collaborative approaches, particularly in the LTC and pandemic context. A recent realist-inspired systematic review^8^ explored the latest evidence on contextual factors and mechanisms impacting the outcome of the QI collaborative approaches. This review study^8^ found that adequate external support, leadership, QI capacity and alignment with systemic pressures and incentives could influence outcome, which was somewhat consistent with our findings. However, it is noteworthy that none of the included studies in the review^8^ were related to pandemic preparedness or involving the LTC sector.

The team-based nature of PAPLOC was found to be an important success factor. Participants appreciated the diversity of the QI team members and opportunities to contribute and provide input. Previous research has also emphasised the importance of establishing multidisciplinary QI teams that give voice to a wide range of perspectives.^23 24^ LTC homes were provided with the flexibility to assemble their own QI teams, but were strongly encouraged to include a resident/family caregiver, which resulted in 4/6 QI teams having a resident/family caregiver member. Resident/family caregiver QI team members provided an experiential perspective that led to unique, useful change ideas. Agenda-setting exercises with LTC residents have previously revealed that often, things that staff may perceive as trivial are what residents believe can improve their quality of life in LTC.^25^ Participation in PAPLOC provided resident/family caregivers an insiders’ perspective on LTC operations, including new insights into staff challenges. Diversity of perspectives could have been enhanced through prioritising inclusion of care aides since several participants noted that their QI teams were top-heavy. However, staff were challenged in making time for QI and felt their hands were tied by external forces during the pandemic.

The PAPLOC approach benefitted participants through empowerment and instilling an appetite for change. PAPLOC has served as a springboard for ongoing, expanding LTC QI initiatives and infrastructure in the FHA region beyond the pandemic. Facilitator supports was a key enabler for ongoing QI work in the LTC homes during the study. This is consistent with lessons from international QI collaboratives advising to recruit QI facilitators with pre-existing relationships in LTC.^12^ Also, the key need to provide sufficient resources to support collecting, processing and interpreting QI data. PAPLOC was conceptualised as flexible to accommodate the dynamic pandemic circumstances and align with local pandemic-related priorities of each LTC home. These pandemic-related facets of PAPLOC have been found by other QI collaboratives as generally relevant within the LTC context irrespective of pandemic-times due to the organisational complexities and limited evidence-base specific to the sector.^12^

Participants felt ambivalent towards the virtual components of PAPLOC. Zoom-based QI meetings were generally effective despite some preference for in-person meetings. Other studies have also found that there are both pros and cons to virtual QI training, and blended in-person and online approaches may be the most valuable to participants.^6^ In the future, it may be beneficial to hold an initial or occasional in-person meeting between the QI facilitators and QI team, which would also provide an opportunity to strengthen rapport. A UK QI collaborative study in care homes found that on-site, in-person meetings enabled staff control within existing routines and uninterrupted participation of external members, for example, physicians.^23^

Slack use was negligible in PAPLOC due to various barriers such as inconvenience and lack of accessibility. Its use across all participating LTC homes was hampered by variable levels of comfort and lack of buy-in and time. These findings are consistent with previous studies on the use of Slack in clinical groups which also identified the availability of multiple platforms/alternative means of communication as a barrier and that having fewer alternative communication options can encourage the use of Slack.^26^ Another challenge that emerged in the PAPLOC study included lack of accessibility for (a) residents with disabilities that prevented them from using Slack or (b) staff who were unable to regularly access a computer. An important lesson from this experience is that in a team-based intervention, the inclusivity of the communication methods used is very important. While Slack was ultimately unsuccessful within PAPLOC, it may have useful applications for other interventions where in-person communication is not an option. For example, for the IST and QI facilitators who were working remotely, Slack was an invaluable platform for coordinating the study and QI projects, respectively.

Limitations of this research should be noted. First, some participants did not complete all of the interviews, and the midpoint sample in particular was small. Challenges with scheduling the midpoint interviews occurred due to their timing which coincided with an unusually destructive forest fire season in BC and other challenging circumstances in the LTC homes. However, as the midpoint/final interviews contained the same questions and there was a high response rate to the final interview, we believe the interviews still were able to adequately capture the experiences and perspectives of the participants. Second, while efforts were made to conduct member checks with participants regarding the themes, no feedback was received despite an initial request and a reminder. While the non-response is potentially a sign of implicit agreement with the themes, it would have been beneficial if feedback was received to confirm the themes. Due to the heavy impact of the pandemic compounded by local climate/natural disasters, it was decided to discontinue ongoing requests for feedback from participants. Finally, given the limited timeframe of the study within the demanding context of the pandemic, assessing the sustainability of the PAPLOC approach proved challenging. The authors emphasise that a future undertaking of an extended follow-up of the participating LTC homes could explore sustainability and serve as a foundation for further developments and learning.

## Conclusion

Overall, the PAPLOC approach provided QI teams in LTC homes the facilitation and coaching required to successfully form QI teams, learn about QI methods, and implement pandemic-related QI projects. The success of the virtual QI collaborative approach within a short period and challenging context provides valuable lessons on QI approaches in health emergency circumstances. For example, a multidisciplinary QI team, including resident and family/informal caregivers, provides useful, diverse perspectives to prioritise and guide the most timely and impactful QI projects. Also, customising QI tools, resources and supports for the LTC sector is key for success, including flexible approaches customised to the needs of LTC homes. Facilitation for QI teams helps keeps QI teams on track and was feasible virtually via video conferencing through Zoom. Although there was limited engagement of QI team members on Slack, the virtual communication platform was instead used primarily by the IST and QI facilitators to coordinate the study and QI projects, respectively.

## Data Availability

All data relevant to the study are included in the article or uploaded as supplementary information.
