# Supplementary material for: Quality improvement collaborative approach to COVID-19 pandemic preparedness in long-term care homes: a mixed-methods implementation study
Source: BMJ Open Qual. 2024 Apr 8;13(2):e002589. doi: 10.1136/bmjoq-2023-002589 (PMC11015329; doi:10.1136/bmjoq-2023-002589)
Supplement: Supplementary data [file bmjoq-2023-002589supp002.pdf]

**Follow-up Interviews (completion/month 9)**

Thank you for participating in the quality improvement team at <<name of long-term care home>>. In this interview, we want to learn more about your experience being involved in the quality improvement activities. Your responses will help to inform ways that healthcare teams can work together virtually to strengthen how prepared long-term care homes are for pandemics. You are welcome to decline responding to any questions or to end this interview at any time. This interview is being conducted by Zoom or by phone. Our conversation will be audio recorded and an anonymized transcript will be produced and assessed collectively with many other interviews. You are welcome to decline responding to any questions or to end this interview at any time. You also have the right to request that a comment be omitted or request that the entire interview be omitted from the study. Do I have your consent to begin?

| Topic / Main Question | Subtopics / Probing Questions |
|---|---|
| Can you tell me about your experience with the quality improvement projects to strengthen pandemic preparedness in your long-term home? | • What has been your role in the Quality Improvement team?<br>• How often have you interacted with your QI team members?<br>• Could you tell me about:<br>o how well the QI projects are progressing?<br>o challenges in the QI projects?<br>o success of the QI projects? |
| On a scale from 1 to 5, where 1 is not at all engaged and 5 is highly engaged, how would you rate your level of engagement on the Quality Improvement team? | If participant has completed a midpoint interview ask:<br>• What, if anything, has changed about your level of engagement on the QI team since our last interview?<br>• What do you think is working well / can be improved in how team members are engaged in QI projects?<br>• What have you learned from and perhaps used from QI projects completed at other LTC homes?<br>• Reflections on any other aspects of the QI collaborative approach. |



Sorensen J, *et al. BMJ Open Qual* 2024; 13:e002589. doi: 10.1136/bmjoq-2023-002589

| | |
|---|---|
| | If participant has not completed a midpoint interview ask:<br>• How do you feel about the way QI is being approached/facilitated in your organization?<br>• How has the QI facilitation team supported the QI process?<br>• Is everyone engaged in this process that should be engaged?<br>• Do you feel you are allowed sufficient opportunity to contribute to QI?<br>• What do you think is working well / can be improved in how team members are engaged in QI projects?<br>• What have you learned from and perhaps used from QI projects completed at other LTC homes? |
| I am going to ask you about your skill level with QI. On a scale from 1 to 5, where 1 is not at all skilled and 5 is highly skilled, how would you rate your skill level doing QI?<br><br>Next, how about your level of confidence with QI. On a scale from 1 to 5, where 1 is not at all confident and 5 is highly confident, how would you rate your level of confidence with QI?<br><br>Lastly, how about your view on the importance of QI. On a scale from 1 to 5, where 1 is not at all important and 5 is highly important, how would you rate the level of importance of QI? | |
| I am going to ask you about how satisfied you have been with the quality improvement initiatives to strengthen pandemic preparedness. On a scale from 1 to 5, with 1 being not at all satisfied and | • Why do you rate your satisfaction at this level?<br>• What has impacted your satisfaction (since the last interview)? |



Sorensen J, *et al. BMJ Open Qual* 2024; 13:e002589. doi: 10.1136/bmjoq-2023-002589

| | |
|---|---|
| 5 being highly satisfied, how satisfied are you? | |
| How has your experience been with the online communication platform Slack to support the quality improvement work? | If participant completed a midpoint interview ask:<br>• What, if anything, has changed about your use of the platform since the last interview?<br>• What other modes of communication have you been using?<br><br>If participant has not completed a midpoint interview ask:<br>• What do you like least and why?<br>• What do you like most and why?<br>• What could be changed to improve your experience?<br>• Could you tell me about your opinion on:<br>    o ease of use<br>    o motivation to use<br>    o challenges in use<br>    o the suitability for using Slack in quality improvement and to support shared learning<br>    o preferences vs. face-to-face communication<br>• If you have not been using the virtual collaborative, please tell us what has prevented you from using it.<br>• Are there other ways to communicate that you have been using to work on QI projects (e.g., Zoom, face-to-face meetings, etc.)? |
| I am going to present you with some statements about joy in work. On a scale from 1 to 5, please tell me how you agree with the following statements, with 1 | Do you believe participation in the QI team at you LTC home has changed your joy in work? If yes, how and why has it changed it? |

| | |
|---|---|
| being strongly disagree and 5 being strongly agree?<br><br>• Being on the QI teams makes me feel like I am part of something meaningful.<br>• I enjoy being a part of the QI team. | |
| I am going to ask you about how satisfied you have been with the QI collaborative approach used in this study to strengthen pandemic preparedness that we've just discussed. On a scale from 1 to 5, with 1 being not at all satisfied and 5 being highly satisfied, how satisfied are you? | • Why do you rate your satisfaction at this level?<br>• What has impacted your satisfaction (since the last interview)?<br>• What have been the most useful aspects of the QI collaborative approach? |
| How do you think the QI collaborative can continue and have a lasting effect after the completion of this study? | • Do you want to continue to participate in your QI team after the study ends?<br>• What might help or prevent the QI team at your home from continuing to work on QI after the study ends?<br>• What type of ongoing support would you like to be made available to your QI team? |
| *Facility Medical Director Question:* Has the care home had COVID-19 outbreak(s) since our last interview? | If yes:<br>• How many outbreaks?<br>• What was the length of the outbreak?<br>• What was the infection and mortality rate? |



Sorensen J, *et al. BMJ Open Qual* 2024; 13:e002589. doi: 10.1136/bmjoq-2023-002589
