# Supplementary material for: Quality improvement collaborative approach to COVID-19 pandemic preparedness in long-term care homes: a mixed-methods implementation study
Source: BMJ Open Qual. 2024 Apr 8;13(2):e002589. doi: 10.1136/bmjoq-2023-002589 (PMC11015329; doi:10.1136/bmjoq-2023-002589)
Supplement: Supplementary data [file bmjoq-2023-002589supp001.pdf]

A quality improvement collaborative approach to COVID-19 pandemic preparedness in long-term care homes: a mixed-methods implementation study

Table S1. Organisational Readiness for Knowledge Translation (OR4KT) at baselines and final interview (n=22)

	Baseline, n (%)				Final, n (%)			
**Dimensions**	**Disagree**	**Neutral**	**Agree**	**N/A**	**Disagree**	**Neutral**	**Agree**	**N/A**
**1) Organizational climate for change**	12 (5%)	43 (20%)	165 (75%)	0 (0%)	14 (6%)	26 (12%)	180 (82%)	0 (0%)
staff work together as a team	0 (0%)	6 (27%)	16 (73%)	0 (0%)	0 (0%)	0 (0%)	22 (100%)	0 (0%)
staff are usually quick to help one another when needed	1(5%)	2 (9%)	19 (86%)	0 (0%)	0 (0%)	2 (9%)	20 (91%)	0 (0%)
mutual trust among staff is strong	0 (0%)	4 (18%)	15 (82%)	0 (0%)	1 (5%)	3 (14%)	18 (82%)	0 (0%)
heavy workload reduces intervention effectiveness	3 (14%)	4 (18%)	15 (68%)	0 (0%)	3 (14%)	5 (23%)	14 (64%)	0 (0%)
staff frustration is common	5 (23%)	8 (36%)	9 (41%)	0 (0%)	7 (32%)	4 (18%)	11 (50%)	0 (0%)
ideas and suggestions from staff get fair consideration by senior leaders	0 (0%)	2 (9%)	20 (91%)	0 (0%)	0 (0%)	5 (23%)	17 (77%)	0 (0%)
formal communication channels work very well	1 (5%)	4 (18%)	15 (68%)	0 (0%)	2 (9%)	5 (23%)	15 (68%)	0 (0%)
staff members feel free to ask questions and express concerns	1 (5%)	4 (18%)	17 (77%)	0 (0%)	0 (0%)	1 (5%)	21 (96%)	0 (0%)
managers are open to staff ideas for improving change	0 (0%)	5 (23%)	17 (77%)	0 (0%)	0 (0%)	1 (5%)	21 (96%)	0 (0%)
managers encourage trying new and different practices	1 (5%)	4 (18%)	17 (77%)	0 (0%)	1 (5%)	0 (0%)	21 (96%)	0 (0%)
**2) Organizational contextual factors**	16 (7%)	25 (11%)	170 (77%)	9 (4%)	13 (6%)	21 (10%)	177 (80%)	9 (4%)
roles and responsibilities are considered as part of the planning process in relation to specific change initiatives	3 (14%)	4 (18%)	15 (68%)	0 (0%)	0 (0%)	1 (5%)	21 (96%)	0 (0%)
has necessary support in terms of budget or financial resources to facilitate change	0 (0%)	7 (32%)	14 (64%)	1 (5%)	2 (9%)	3 (14%)	15 (68%)	2 (9%)
has necessary support in terms of training to facilitate change	1 (5%)	0 (0%)	21 (96%)	0 (0%)	2 (9%)	2 (9%)	16 (73%)	2 (9%)
has necessary support in terms of facilities and equipment to facilitate change	4 (18%)	3 (14%)	14 (64%)	1 (5%)	0 (0%)	1 (5%)	21 (96%)	0 (0%)
has necessary support in terms of staffing numbers to facilitate change	2 (9%)	3 (14%)	16 (73%)	1 (5%)	5 (23%)	5 (23%)	12 (55%)	0 (0%)
managers solicit opinions of clinical staff regarding decisions about patient care	0 (0%)	0 (0%)	22 (100%)	0 (0%)	0 (0%)	1 (5%)	21 (96%)	0 (0%)
staff members have a sense of personal responsibility for improving patient care and outcomes	0 (0%)	0 (0%)	22 (100%)	0 (0%)	1 (5%)	1 (5%)	20 (91%)	0 (0%)
staff members cooperate to maintain and improve effectiveness of patient care	4 (18%)	2 (9%)	16 (73%)	0 (0%)	0 (0%)	2 (9%)	20 (91%)	0 (%)
staff members are willing to innovate and/or experiment to improve clinical procedures	2 (9%)	6 (27%)	14 (64%)	0 (0%)	1 (5%)	3 (14%)	13 (59%)	5 (23%)

Sorensen J, *et al. BMJ Open Qual* 2024; 13:e002589. doi: 10.1136/bmjoq-2023-002589

A quality improvement collaborative approach to COVID-19 pandemic preparedness in long-term care homes: a mixed-methods implementation study

	Baseline, n (%)				Final, n (%)			
**Dimensions**	**Disagree**	**Neutral**	**Agree**	**N/A**	**Disagree**	**Neutral**	**Agree**	**N/A**
staff members are receptive to change in clinical processes	0 (0%)	6 (27%)	16 (73%)	0 (0%)	2 (9%)	2 (9%)	18 (82%)	0 (0%)
**3) Change content**	5 (3%)	44 (16%)	146 (65%)	3 (2%)	7 (4%)	33 (12%)	158 (80%)	0 (0%)
there is willingness to adjust to changes	0 (0%)	7 (32%)	15 (68%)	0 (0%)	2 (9%)	11 (50%)	9 (41%)	0 (0%)
there is ability to exchange ideas and influence decisions related to delivery of patient care	0 (0%)	5 (23%)	17 (77%)	0 (0%)	1 (5%)	1 (5%)	20 (91%)	0 (0%)
there is flexibility to deal with change	1 (5%)	2 (9%)	19 (86%)	0 (0%)	0 (0%)	2 (9%)	20 (91%)	0 (0%)
people are willing to adjust usual routines in response to what is happening around them	3 (14%)	1 (5%)	18 (82%)	0 (0%)	2 (9%)	6 (27%)	14 (64%)	0 (0%)
typically there is adaptability to new standards or procedures, even those forced upon us	0 (0%)	2 (9%)	20 (91%)	0 (0%)	0 (0%)	6 (27%)	16 (73%)	0 (0%)
proposed changes have been well accepted by patients prior to implementation (e.g., in a pilot study)	1 (5%)	8 (36%)	10 (46%)	3 (14%)	1 (5%)	3 (14%)	18 (82%)	0 (0%)
proposed changes take into consideration the needs and preferences of patients	0 (0%)	6 (27%)	16 (73%)	0 (0%)	0 (0%)	2 (9%)	20 (91%)	0 (0%)
proposed changes appear to have more advantages than disadvantages for patients	0 (0%)	5 (23%)	17 (77%)	0 (0%)	0 (0%)	2 (9%)	20 (91%)	0 (0%)
proposed changes should be effective, based on current scientific knowledge	0 (0%)	8 (36%)	14 (64%)	0 (0%)	1 (5%)	0 (0%)	21 (96%)	0 (0%)
**4) Leadership**	15 (7%)	42 (19%)	161 (73%)	2 (1%)	11 (5%)	25 (11%)	175 (80%)	9 (4%)
managers provide effective management for continuous improvement of patient care	0 (0%)	2 (9%)	20 (91%)	0 (0%)	0 (0%)	2 (9%)	20 (91%)	0 (0%)
managers provide staff members with feedback/data on effects of clinical decisions	0 (0%)	3 (14%)	19 (86%)	0 (0%)	1 (5%)	1 (5%)	20 (91%)	0 (0%)
managers hold staff members accountable for achieving results	0 (0%)	8 (36%)	14 (64%)	0 (0%)	0 (0%)	2 (9%)	20 (91%)	0 (0%)
external stakeholders are involved in the planning process	2 (9%)	5 (23%)	13 (59%)	2 (9%)	4 (18%)	1 (5%)	14 (64%)	3 (14%)
all staff members are usually included in decision-making processes	8 (36%)	1 (5%)	13 (59%)	0 (0%)	3 (14%)	7 (32%)	12 (55%)	0 (0%)
there is an innovation decision- maker on key organizational clinical committees	2 (9%)	8 (36%)	12 (55%)	0 (0%)	1 (5%)	2 (9%)	19 (86%)	0 (0%)
there is an innovation decision- maker on key organizational administrative committees	2 (9%)	7 (32%)	13 (59%)	0 (0%)	1 (5%)	1 (5%)	17 (77%)	3 (14%)
managers are involved in the change process	0 (0%)	0 (0%)	22 (100%)	0 (0%)	0 (0%)	2 (9%)	20 (91%)	0 (0%)
clinicians are involved in the change process	0 (0%)	0 (0%)	22 (100%)	0 (0%)	0 (0%)	2 (9%)	19 (86%)	1 (5%)
administrative and clerical staff are involved in the change process	1 (5%)	8 (36%)	13 (59%)	0 (0%)	1 (5%)	5 (23%)	14 (64%)	2 (9%)

Sorensen J, *et al. BMJ Open Qual* 2024; 13:e002589. doi: 10.1136/bmjoq-2023-002589

A quality improvement collaborative approach to COVID-19 pandemic preparedness in long-term care homes: a mixed-methods implementation study

	Baseline, n (%)				Final, n (%)			
**Dimensions**	**Disagree**	**Neutral**	**Agree**	**N/A**	**Disagree**	**Neutral**	**Agree**	**N/A**
**5) Organizational support**	17 (8%)	50 (23%)	153 (70%)	0 (0%)	13 (6%)	24 (11%)	181 (82%)	2 (1%)
team members provide practical support for new ideas & their application	0 (0%)	6 (27%)	16 (73%)	0 (0%)	0 (0%)	2 (9%)	20 (91%)	0 (0%)
assistance in developing new ideas is readily available	5 (23%)	3 (14%)	14 (64%)	0 (0%)	2 (9%)	3 (14%)	17 (77%)	0 (0%)
team members cooperate in order to help develop and apply new ideas	0 (0%)	4 (18%)	18 (82%)	0 (0%)	0 (0%)	0 (0%)	22 (100%)	0 (0%)
team members provide and share resources to help in the application of new ideas	0 (0%)	8 (36%)	14 (64%)	0 (0%)	1 (5%)	2 (9%)	19 (86%)	0 (0%)
the change process is monitored continuously	6 (28%)	5 (23%)	11 (50%)	0 (0%)	2 (9%)	3 (14%)	17 (77%)	0 (0%)
outcomes are monitored continuously	1 (5%)	3 (14%)	18 (82%)	0 (0%)	2 (9%)	3 (14%)	17 (77%)	0 (0%)
periodic outcome measurements are used to evaluate and improve implementation of planned changes	2 (9%)	2 (9%)	18 (82%)	0 (0%)	1 (5%)	3 (14%)	18 (82%)	0 (0%)
dissemination of performance measures to key stakeholders is used to evaluate and improve implementation of planned changes	1 (5%)	11 (50%)	10 (45%)	0 (0%)	2 (9%)	5 (23%)	13 (59%)	2 (9%)
leaders review results to evaluate and improve planned changes	0 (0%)	3 (14%)	19 (86%)	0 (0%)	1 (5%)	0 (0%)	21 (95%)	0 (0%)
there are formal mechanisms established for obtaining feedback related to the proposed change	2 (9%)	5 (23%)	15 (68%)	0 (0%)	2 (9%)	3 (14%)	17 (77%)	0 (0%)
**6) Motivation**	19 (9%)	61 (28%)	136 (62%)	4 (2%)	17 (8%)	33 (15%)	164 (75%)	6 (3%)
pressure to make change comes from patients	7 (32%)	7 (32%)	8 (36%)	0 (0%)	7 (32%)	4 (18%)	11 (50%)	0 (0%)
pressure to make change come from staff members	4 (18%)	12 (55%)	6 (27%)	0 (0%)	0 (0%)	10 (45%)	12 (55%)	0 (0%)
pressure to make change comes from senior leaders	1 (5%)	5 (23%)	14 (64%)	2 (9%)	0 (0%)	4 (18%)	18 (82%)	0 (0%)
pressure to make change comes from board members or overseers	3 (14%)	12 (55%)	6 (27%)	1 (5%)	2 (9%)	5 (23%)	13 (59%)	2 (9%)
pressure to make change comes from funding organizations	1 (5%)	2 (9%)	19 (86%)	0 (0%)	3 (14%)	3 (14%)	14 (64%)	2 (9%)
experience has been gained in implementing change from projects or pilot programs and their evaluation	0 (0%)	4 (18%)	17 (77%)	1 (5%)	1 (5%)	0 (0%)	21 (95%)	0 (0%)
managers are knowledgeable about innovation based on their past experience	0 (0%)	8 (36%)	14 (64%)	0 (0%)	0 (0%)	3 (14%)	19 (86%)	0 (0%)
knowledge is available about how similar innovations are being used by other organizations	3 (14%)	5 (23%)	14 (64%)	0 (0%)	2 (9%)	1 (5%)	19 (86%)	0 (0%)
senior managers promote change by behaving in a consistent manner	0 (0%)	2 (9%)	20 (91%)	0 (0%)	1 (5%)	1 (5%)	18 (82%)	2 (9%)
senior managers define the course of change	0 (0%)	4 (18%)	18 (82%)	0 (0%)	1 (5%)	2 (9%)	19 (86%)	0 (0%)
